# About the complexity of two-stage stochastic IPs

**DOI:** 10.1007/s10107-021-01698-z

**Published:** 2021-09-08

**Authors:** Kim-Manuel Klein

**Affiliations:** University of Kiel, Kiel, Germany

**Keywords:** Integer programming, Parameterized compexity, Two-stage stochastic, Stochastic programming, 90C10, 90C15

## Abstract

We consider so called 2-stage stochastic integer programs (IPs) and their generalized form, so called multi-stage stochastic IPs. A 2-stage stochastic IP is an integer program of the form $\max \{ c^T x \mid {\mathcal {A}} x = b, \,l \le x \le u,\, x \in {\mathbb {Z}}^{s + nt} \}$ where the constraint matrix ${\mathcal {A}} \in {\mathbb {Z}}^{r n \times s +nt}$ consists roughly of *n* repetitions of a matrix $A \in {\mathbb {Z}}^{r \times s}$ on the vertical line and *n* repetitions of a matrix $B \in {\mathbb {Z}}^{r \times t}$ on the diagonal. In this paper we improve upon an algorithmic result by Hemmecke and Schultz from 2003 [Hemmecke and Schultz, Math. Prog. 2003] to solve 2-stage stochastic IPs. The algorithm is based on the Graver augmentation framework where our main contribution is to give an explicit doubly exponential bound on the size of the augmenting steps. The previous bound for the size of the augmenting steps relied on non-constructive finiteness arguments from commutative algebra and therefore only an implicit bound was known that depends on parameters *r*, *s*, *t* and $\Delta$, where $\Delta$ is the largest entry of the constraint matrix. Our new improved bound however is obtained by a novel theorem which argues about intersections of paths in a vector space. As a result of our new bound we obtain an algorithm to solve 2-stage stochastic IPs in time $f(r,s,\Delta ) \cdot \mathrm {poly}(n,t)$, where *f* is a doubly exponential function. To complement our result, we also prove a doubly exponential lower bound for the size of the augmenting steps.

## Introduction

Integer programming is one of the most fundamental problems in algorithm theory. Many problems in combinatorial optimization and other areas can be modeled by integer programs. An *integer program* (IP) is thereby of the form

$$\begin{aligned} \max \{ c^T x \mid Ax = b, \, l \le x \le u, \, x \in {\mathbb {Z}}^n \} \end{aligned}$$

for some matrix $A \in {\mathbb {Z}}^{m \times n}$, a right hand side $b \in {\mathbb {Z}}^m$, a cost vector $c \in {\mathbb {Z}}^n$ and lower and upper bounds $l, u \in {\mathbb {Z}}^n$. The famous algorithm of Kannan [22] computes an optimal solution of the IP in time of roughly $n^{O(n)} \cdot \mathrm {poly}(m,\log \Delta )$, where $\Delta$ is the largest entry of *A* and *b*.

In recent years there was significant progress in the development of algorithms for IPs when the constraint matrix *A* has a specific structure. Consider for example the class of integer programs with a constraint matrix ${\mathcal {N}}$ of the form

$$\begin{aligned} {\mathcal {N}} = \begin{pmatrix} A &{} A &{} \cdots &{} A \\ B &{} 0 &{} \cdots &{} 0\\ 0 &{} B &{} \ddots &{} \vdots \\ \vdots &{} \ddots &{} \ddots &{} 0 \\ 0 &{} \cdots &{} 0 &{} B \end{pmatrix} \end{aligned}$$

for some matrices $A \in {\mathbb {Z}}^{r \times s}$ and $B \in {\mathbb {Z}}^{r \times t}$. An IP of this specific structure is called an *n*-*fold* IP. This class of IPs has found numerous applications in the area of string algorithms [24], computational social choice [25] and scheduling [19, 23]. State-of-the-art algorithms compute a solution of an *n*-fold IP in time $\Delta ^{O(r^2s+s^2)} \cdot (n,t)^{1+o(1)}$ [7, 10, 11, 20, 26], where $\Delta$ is the largest entry in the matrices *A* and *B*.

### Two-stage stochastic integer programming

Stochastic programming deals with uncertainty of decision making over time [21]. One of the basic models in stochastic programming is 2-stage stochastic programming. In this model one has to decide on a solution at the first stage and in the second stage there is an uncertainty where *n* possible scenarios can happen. Each of *n* possible scenarios might have a different optimal solution and the goal is to minimize the costs of the solution of the first stage in addition to the expected costs of the solution of the second stage. In the case that said scenarios can be modeled by an (integer) linear program, we are talking about 2-*stage stochastic (integer) linear programs*. 2-stage stochastic linear programs that do not contain any integer variable are well understood (we refer to standard text books [3, 21]). In contrast, 2-stage stochastic programs that contain integer variables are hard to solve and are the topic of ongoing research. Typically, those IPs are investigated in the context of decomposition based methods (we refer to a tutorial [27] or a survey [31] on the topic). For progress on 2-stage stochastic programs we refer to [1, 5, 31]. The interest in solving 2-stage stochastic (I)LPs efficiently stems from their wide range of applications for example in modeling manufacturing processes [9] or energy planing [17].

In this paper we consider 2-stage stochastic IPs with only integral variables. For the extension of the results of this paper to the mixed setting we refer to [4]. So called pure integral 2-stage stochastic IPs have also been considered in the literature from a practical perspective (see [14, 33]). The considered IP is then of the form

1 $$\begin{aligned}&\max c^T x \nonumber \\&{\mathcal {A}}x = b\nonumber \\&l \le x \le u\nonumber \\&x \in {\mathbb {Z}}^{s + nt} \end{aligned}$$

for given objective vector $c \in {\mathbb {Z}}^{s + nt}$, upper and lower bound $l, u \in {\mathbb {Z}}^{s + nt}$. The constraint matrix ${\mathcal {A}}$ has the shape

$$\begin{aligned} {\mathcal {A}} = \begin{pmatrix} A^{(1)} &{} B^{(1)} &{} 0 &{} \cdots &{} 0\\ A^{(2)} &{} 0 &{} B^{(2)} &{} \ddots &{} \vdots \\ \vdots &{} \vdots &{} \ddots &{} \ddots &{} 0 \\ A^{(n)} &{} 0 &{} \cdots &{} 0 &{} B^{(n)} \end{pmatrix} \end{aligned}$$

for given matrices $A^{(1)}, \ldots , A^{(n)} \in {\mathbb {Z}}^{r \times s}$ and $B^{(1)}, \ldots , B^{(n)} \in {\mathbb {Z}}^{r \times t}$.

Typically, 2-stage stochastic IPs are written in a slightly different (equivalent) form that explicitly involves the scenarios and the probability distribution of the scenarios of the second stage. In this presented form, roughly speaking, the solution for the first stage scenario is encoded in the variables corresponding to vertical matrices. A solution for each of the second stage scenarios is encoded in the variables corresponding to one of the diagonal matrices and the expectation for the second stage scenarios can be encoded in a linear objective function. Since we do not rely on known techniques of stochastic programming in this paper, we omit the technicalities surrounding 2-stage stochastic IPs and simply refer to a survey for further details [31].

Despite their similarity, it seems that 2-stage IPs are significantly harder to solve than *n*-fold IPs. While Hemmecke and Schultz [18] have shown that a 2-stage stochastic IP with constraint matrix ${\mathcal {A}}$ can be solved in running time of the form $f(r,s,t, \Delta ) \cdot \mathrm {poly}(n)$ for some computable function *f*, the actual dependence on the parameters $r,s,t, \Delta$ was unknown (we elaborate on this further in the coming section). Their algorithm is based on the augmentation framework which we also discuss in the following section.

### Graver elements and the augmentation framework

Suppose we have an initial feasible solution $z_0$ of an IP $\max \{ c^T x \mid Ax = b, \, l \le x \le u, \, x \in {\mathbb {Z}}^n \}$ and our goal is to find an optimal solution. The idea behind the augmentation framework (see [11]) is to compute an augmenting (integral) vector *y* in the kernel, i.e., $y \in \mathrm {ker}(A)$ with $c^T y > 0$. A new solution $z'$ with improved objective can then be defined by $z' = z_0 + \lambda y$ for a suitable $\lambda \in {\mathbb {Z}}_{>0}$. This procedure can be iterated until a solution with optimal objective is obtained eventually.

We call an integer vector $y \in \mathrm {ker}(A)$ a *cycle*. A cycle can be decomposed if there exist integral vectors $u,v \in \mathrm {ker}(A){\setminus } \{ 0 \}$ with $y = u + v$ and $u_i \cdot v_i \ge 0$ for all *i* (i.e., the vectors are sign-compatible with *y*). An integral vector $y \in \mathrm {ker}(A) {\setminus } \{ 0\}$ that can not be decomposed is called a *Graver element* [15] or we simply say that it is *indecomposable*. The set of all indecomposable elements is called the *Graver basis*.

For a given bound on the size of Graver elements of the constraint matrix, an augmenting vector $y \in \mathrm {ker}(A)$ can often be computed by a dynamic program (depending on the structure of the constraint matrix), whereas the running time of the dynamic program depends on the respective bound. An optimal solution of the corresponding IP can then be solved by using the augmentation framework. For a detailed description of the augmentation framework we refer to the paper by Eisenbrand et al. [11].

In the case that the constraint matrix has a very specific structure, one can sometimes show improved bounds. Specifically, if the constraint matrix *A* has a 2-stage stochastic shape with identical matrices in the vertical and diagonal line, then Hemmecke and Schultz [18] were able to prove a bound for the size of Graver elements that only depends on the parameters *r*, *s*, *t* and $\Delta$. The presented bound is an existential result and uses so called saturation results from commutative algebra. In their line of proof MacLagan’s theorem is used, which only yields a finiteness statement (i.e., there are no infinite antichains in the set of monomial ideals in a polynomial ring in finitely many variables over a field) and no explicit bound is known yet for this quantity. It is only known that the dependence on the parameters is lower bounded by Ackerman’s function [28]. This implies that the parameter dependence of *r*, *s*, *t* and $\Delta$ in the implicit bound of the size of Graver elements by Hemmecke and Schultz is at least ackermanian.

Very recently, improved bounds for Graver elements of general matrices and matrices with specific structure like *n*-fold [10] or 4-block structure [6] were developed.

#### Lemma 1

(Steinitz [16, 32]) Let $v_1, \ldots , v_n \in {\mathbb {R}}^m$ be vectors with $\left\| v_i\right\| _{\infty } \le \Delta$ for $1 \le i \le n$. Assuming that $\sum _{i=1}^n v_i = 0$ then there is a permutation $\pi$ such that for each $k \in \{1, \ldots , n \}$ the norm of the partial sum $\left\| \sum _{i=1}^k v_{\pi (i)}\right\| _{\infty }$ is bounded by $m \Delta$

The Steinitz Lemma was used by Eisenbrand, Hunkenschröder and Klein [10] to bound the size of Graver elements for a given matrix *A*. As we use the following theorem and its technique in this paper, we give a brief sketch of its proof. The Steinitz Lemma was first used by Eisenbrand and Weismantel [12] in the context of integer programming.

#### Theorem 1

(Eisenbrand, Hunkenschröder, Klein [10]) Let $A \in {\mathbb {Z}}^{m \times n}$ be an integer matrix where every entry of *A* is bounded by $\Delta$ in absolute value. Let $g \in {\mathbb {Z}}^n$ be an element of the Graver Basis of *A* then $\left\| g\right\| _1 \le (2m\Delta +1)^m$.

#### Proof

Consider the sequence of vectors $v_1, \ldots , v_{\left\| g\right\| _1}$ consisting of $y_i$ copies of the *i*th column of *A* if $g_i$ is positive and $|g_i|$ copies of the negative of the *i*th coplumn of *A* if $g_i$ is negative. As *g* is a Graver element we obtain that $v_1 + \cdots + v_{\left\| g\right\| _1} = 0$. Using the Steinitz Lemma above, there exists a reordering $u_1 + \cdots + u_{\left\| g\right\| _1}$ of the vectors such that the partial sum $p_k = \left\| \sum _{i=1}^{k} u_i\right\| _{\infty } \le \Delta m$ for each $k \le \left\| g\right\| _1$.

Suppose by contradiction that $\left\| g\right\| _1 > (2m\Delta +1)^m$. Then by the pigeonhole principle there exist two partial sums that sum up to the same value. However, this means that *g* can be decomposed and hence can not be a Graver element. $\square$

### Our results

The main result of this paper is to prove a new structural lemma that enhances the toolset of the augmentation framework. We show that this lemma can be directly used to obtain an explicit bound for Graver elements of the constraint matrix of 2-stage stochastic IPs. But we think that it might also be of independent interest as it provides interesting structural insights into vector sets.

#### Lemma 2

Given are multisets $T_1, \ldots , T_n \subset {\mathbb {Z}}_{\ge 0}^d$ where all elements $t \in T_i$ have bounded size $\left\| t\right\| _{\infty } \le \Delta$. Assuming that the total sum of all elements in each set is equal, i.e.,

$$\begin{aligned} \sum _{t \in T_1} t = \ldots = \sum _{t \in T_n} t \end{aligned}$$

then there exist nonempty submultisets $S_1 \subseteq T_1, \ldots , S_n \subseteq T_n$ of bounded size $|S_i| \le (d \Delta )^{O(d (\Delta ^{d^2}))}$ such that

$$\begin{aligned} \sum _{s \in S_1} s = \ldots = \sum _{s \in S_n} s. \end{aligned}$$

Note that Lemma 2 only makes sense when we consider the $T_i$ to be multisets as the number of different sets without allowing multiplicity of vectors would be bounded by $2^{(\Delta +1)^d}$.

A geometric interpretation of Lemma 2 is given in the following figure. On the left side we have *n* paths consisting of sets of vectors and all paths end at the same point *b*.


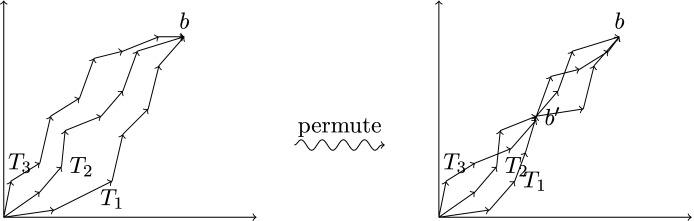


Then Lemma 2 shows that there always exist permutations of the vectors of each path such that all paths meet at a point $b'$ of bounded size. The bound depends only on $\Delta$ and the dimension *d* and is thus independent of the number of paths *n* and the size of *b*. For the Proof of Lemma 2 we need basic properties of the intersections of integer cones. We show that those properties can be obtained by using the Steinitz Lemma.

We show that Lemma 2 has strong implications in the context of integer programming. Using Lemma 2, we can show that the size of Graver elements of the matrix ${\mathcal {A}}$ is bounded by $(r s \Delta )^{O(r s ((2 r \Delta +1)^{r s^2}))}$. Within the framework of Graver augmenting steps the bound implies that 2-stage stochastic IPs can be solved in time $(r s \Delta )^{O(r s^2 ((2 r \Delta +1)^{r s^2}))} \cdot n^2t^2 \varphi \log ^2 (nt)$, where $\varphi$ is the encoding length of the instance (see Theorem 3). With this we improve upon an implicit bound for the size of the Graver elements matrix 2-stage stochastic constraint matrices due to Hemmecke and Schultz [18].

Based on the structural observations of this paper, in a recent work by Cslovjecsek et al. [8] an algorithm was developed that solves 2-stage stochastic IPs with an improved running time of $2^{2\Delta ^{O(r(r+s))}} \cdot n \log ^{O(rs)}n$.

Furthermore, we show that our Lemma can also be applied to bound the size of Graver elements of constraint matrices that have a multi-stage stochastic structure. Multi-stage stochastic IPs are a well known generalization of 2-stage stochastic IPs. By this, we improve upon a result of Aschenbrenner and Hemmecke [2].

To complement our results for the upper bound, we also present in Sect. 3 a lower bound for the size of Graver elements of matrices that have a 2-stage stochastic IP structure. The given lower bound is for the case of $s=1$. In this case we show in Theorem 4 a matrix where the Graver elements have a size of $2^{\Omega (\Delta ^r)}$.

## The complexity of two-stage stochastic IPs

First, we argue about the application of Lemma 2. In the following we show that the infinity-norm of Graver elements of matrices with a 2-stage stochastic structure can be bounded using Lemma 2.

Given the block structure of the IP (1), we define for a vector $y \in {\mathbb {Z}}^{s + nt}$ with ${\mathcal {A}}y = 0$ the vector $y^{(0)} \in {\mathbb {Z}}_{\ge 0}^{s}$ which consists of the entries of *y* that belong to the vertical matrices $A^{(i)}$ and we define $y^{(i)} \in {\mathbb {Z}}_{\ge 0}^t$ to be the entries of *y* that belong to the diagonal matrix $B^{(i)}$.

### Theorem 2

Let *y* be a Graver element of the constraint matrix ${\mathcal {A}}$ of IP (1). Then $\left\| y\right\| _{\infty }$ is bounded by $(r s \Delta )^{O(r s ((2 r \Delta +1)^{r s^2}))}$. More precisely, $\left\| y^{(i)}\right\| _1 \le (r s \Delta )^{O(r s ((2 r \Delta +1)^{r s^2}))}$ for every $0 \le i \le n$.

### Proof

Let $y \in {\mathbb {Z}}^{s+nt}$ be a cycle of IP (1), i.e., ${\mathcal {A}} y = 0$. Consider a submatrix $(A^{(i)} \, B^{(i)}) \in {\mathbb {Z}}^{r \times (s+t)}$ of the matrix ${\mathcal {A}}$ denoted by consisting of the matrix $A^{(i)}$ of the vertical line and the matrix $B^{(i)}$ of the diagonal line. Consider further the corresponding variables $$v^{(i)} = \begin{pmatrix} y^{(0)}\\ y^{(i)} \end{pmatrix} \in {\mathbb {Z}}^{s+t}$$ of the respective matrix $A^{(i)}$ and $B^{(i)}$. Since ${\mathcal {A}}y = 0$, we also have that $( A^{(i)} B^{(i)} ) v^{(i)} = 0$. By using Theorem 1 iteratively, we can decompose $v^{(i)}$ into a multiset $C_i$ of indecomposable elements, i.e., $v^{(i)} = \sum _{z\in C_i} z$ with $\left\| z\right\| _1 \le (2r \Delta +1)^{r}$ for each $z \in C_i$.

Since all matrices $(A^{(i)} \, B^{(i)})$ share the same set of variables in the overlapping matrices $A^{(i)}$, we can not directly derive cycles for the entire matrix ${\mathcal {A}}$ from cycles of the submatrices $(A^{(i)} \, B^{(i)})$. This is because a cycle $v \in (A^{(i)} \, B^{(i)})$ and a cycle $v'\in (A^{(j)} \, B^{(j)})$ for $j \ne i$ might have conflicting entries in the overlapping part of the vector.

Let $p: {\mathbb {Z}}^{s+t} \rightarrow {\mathbb {Z}}^s$ be the projection that maps a cycle *z* of a block matrix $(A^{(i)} \, B^{(i)})$ to the variables in the overlapping part, i.e., $$p(z) = p(\begin{pmatrix} z^{(0)} \\ z^{(i)} \end{pmatrix}) = z^{(0)}$$.

In the case that $\left\| y\right\| _{\infty }$ is large we will show that we can find a cycle ${\bar{y}}$ of smaller length with $|{\bar{y}}_i| \le |y_i|$ and therefore show that *y* can be decomposed. In order to obtain this cycle ${\bar{y}}$ for the entire matrix ${\mathcal {A}}$, we have to find a multiset of cycles ${\bar{C}}_i \subset C_i$ in each block matrix $(A^{(i)} \, B^{(i)})$ such that the sum of the projected parts is identical, i.e., $\sum _{z \in {\bar{C}}_1} p(z) = \ldots = \sum _{z \in {\bar{C}}_n} p(z)$. We apply Lemma 2 to the multisets $p(C_1), \ldots , p(C_n)$, where $p(C_i) = \{p(z) \mid z \in C_i \}$ is the multiset of projected elements in $C_i$, where $\left\| p(z)\right\| _1 \le (2r \Delta +1)^{r}$ holds. Note that $\sum _{x \in p(C_1)} x = \ldots = \sum _{x \in p(C_n)} x = y^{(0)}$ and hence the conditions to apply Lemma 2 are fulfilled. Since every $v^{(i)}$ is decomposed in a sign compatible way, every entry of the vector in $p(C_i)$ has the same sign. Hence we can flip the negative signs in order to apply Lemma 2.

By Lemma 2, there exist submultisets $S_1 \subseteq p(C_1), \ldots , S_n \subseteq p(C_n)$ such that $\sum _{x \in S_1} x = \ldots = \sum _{x \in S_n} x$ and $|S_i| \le (s \left\| z\right\| _1)^{O(s (\left\| z\right\| _1^{s^2}))} = (r s \Delta )^{O(r s ((2 r \Delta +1)^{r s^2}))}$. As there exist submultisets ${\bar{C}}_1 \subseteq C_1, \ldots {\bar{C}}_n \subseteq C_n$ with $p(\bar{C_1}) = S_1, \ldots p({\bar{C}}_n) = S_n$, we can use those submultisets ${\bar{C}}_i$ to define a solution ${\bar{y}}$ with $|{\bar{y}}_i| \le |y_i|$. For $i>0$ let ${\bar{y}}^{(i)} = \sum _{z \in {\bar{C}}_i} {\bar{p}}(z)$, where ${\bar{p}}(z)$ is the projection that maps a cycle $z \in {\bar{C}}_i$ to the part that belongs to matrix $B^{(i)}$, i.e., $${\bar{p}}(\begin{pmatrix} z^{(0)} \\ z^{(i)} \end{pmatrix}) = z^{(i)}$$. Let ${\bar{y}}^{(0)} = \sum _{z \in {\bar{C}}_i} p(z)$ for an arbitrary $i>0$, which is well defined as the sum is identical for all multisets ${\bar{C}}_i$. As the cardinality of the multisets ${\bar{C}}_i$ is bounded, we know by construction of ${\bar{y}}$ that the one-norm of every $y^{(i)}$ is bounded by

$$\begin{aligned} \left\| y^{(i)}\right\| _1 \le (2r \Delta +1)^{r} \cdot (r s \Delta )^{O(r s ((2 r \Delta +1)^{r s^2}))} = (r s \Delta )^{O(r s ((2 r \Delta +1)^{r s^2}))}. \end{aligned}$$

This directly implies the infinity-norm bound for *y* as well. $\square$

As a consequence of the bound for the size of the Graver elements, we obtain by the framework of augmenting steps an efficient algorithm to compute an optimal solution of a 2-stage stochastic IP. By using the augmentation framework as described in [11] we obtain the following theorem regarding the worst-case complexity for solving 2-stage stochastic IPs.

### Theorem 3

A 2-stage stochastic IP of the form (1) can be solved in time

$$\begin{aligned} n^2t^2 \varphi \log ^2 (nt)(r s \Delta )^{O(r s^2 ((2 r \Delta +1)^{r s^2}))}, \end{aligned}$$

where $\varphi$ is the encoding length of the IP.

### Proof

Let $L = (r s \Delta )^{O(r s ((2 r \Delta +1)^{r s^2}))}$ be the bound for $\left\| y^{(i)}\right\| _1$ that we obtain from the previous Lemma. To find the optimal augmenting step, it is sufficient to solve the so called augmenting IP

2 $$\begin{aligned}&\max c^T x \nonumber \\&{\mathcal {A}}x = 0\nonumber \\&{\bar{l}} \le x \le {\bar{u}}\nonumber \\&\left\| x\right\| _{\infty } \le L \nonumber \\&x \in {\mathbb {Z}}^{s+nt} \end{aligned}$$

for some upper and lower bounds ${\bar{l}}, {\bar{u}}$. Having the best augmenting step at hand, one can show that the objective value improves by a certain factor. We refer to Corollary 14 of [11] which shows that IP (1) can be solved if the above augmenting IP (2) can be solved.

In the following we briefly show how to solve the IP (2) in order to compute the augmenting step. The algorithm works as follows:

- Compute for every $y^{(0)}$ with $\left\| y^{(0)}\right\| _1 \le L$ the objective value of the cycle *y* consisting of $y^{(0)}, {\bar{y}}^{(1)}, \ldots , {\bar{y}}^{(n)}$, where ${\bar{y}}^{(i)}$ for $i>0$ are the optimal solutions of the IP
  $$\begin{aligned} \max&(c^{(i)})^T {\bar{y}}^{(i)} \\ B^{(i)}{\bar{y}}^{(i)}&= - A^{(i)} y^{(0)}\\ {\bar{l}}^{(i)} \le&{\bar{y}}^{(i)} \le {\bar{u}}^{(i)} \end{aligned}$$
  where ${\bar{l}}^{(i)}, {\bar{u}}^{(i)}$ are the upper and lower bounds for the variables ${\bar{y}}^{(i)}$ and $c^{(i)}$ their corresponding objective vector. Note that the first set of constraints of the IP ensure that ${\mathcal {A}}y = 0$. The IPs can be solved with the algorithm of Eisenbrand and Weismantel [12] in time $O(\Delta ^{O(r^2)})$ each.
- Return the cycle with maximum objective.

As the number of different vectors $y^{(0)}$ with 1-norm $\le L$ is bounded by $(L+1)^s = (r s \Delta )^{O(r s^2 ((2 r \Delta +1)^{r s^2}))}$ step 1 of the algorithm takes time $\Delta ^{O(r^2)} \cdot (r s \Delta )^{O(r s^2 ((2 r \Delta +1)^{r s^2}))}$. $\square$

### About the intersection of integer cones

Before we are ready to prove our main Lemma 2, we need two helpful observations about the intersection of integer cones. An integer cone is defined for a given (finite) generating set $B \subset {\mathbb {Z}}_{\ge 0}^d$ of elements by

$$\begin{aligned} \mathrm {int.cone}(B) = \{ \sum _{b \in B} \lambda _b b \mid \lambda \in {\mathbb {Z}}_{\ge 0}^B \}. \end{aligned}$$

Note that the intersection of two integer cones is again an integer cone, as the intersection is closed under addition and scalar multiplication of positive integers.

We say that an element *b* of an integer cone $\mathrm {int.cone}(B)$ is *indecomposable* if there do not exist elements $b_1, b_2 \in \mathrm {int.cone}(B) {\setminus } \{ 0\}$ such that $b = b_1 + b_2$. We can assume that the generating set *B* of an integer cone consists just of the set of indecomposable elements as any decomposable element can be removed from the generating set.

In the following we allow to use a vector set *B* as a matrix and vice versa where the elements of the set *B* are the columns of the matrix *B*. This way we can multiply *B* with a vector, i.e., $B \lambda = \sum _{b \in B} \lambda _b b$ for some $\lambda \in {\mathbb {Z}}^B$.

#### Lemma 3

Consider integer cones $\mathrm {int.cone}(B^{(1)})$ and $\mathrm {int.cone}(B^{(2)})$ for some generating sets $B^{(1)}, B^{(2)} \subset {\mathbb {Z}}^{d}$ where each element $x \in B^{(1)} \cup B^{(2)}$ has bounded norm $\left\| x\right\| _{\infty } \le \Delta$. Consider the integer cone of the intersection

$$\begin{aligned} \mathrm {int.cone}({\hat{B}}) = \mathrm {int.cone}(B^{(1)}) \cap \mathrm {int.cone}(B^{(2)}) \end{aligned}$$

for some generating set of elements ${\hat{B}}$. Then for each indecomposable element $b \in {\hat{B}}$ of the intersection cone with $b = B^{(1)} \lambda = B^{(2)} \gamma$ for some $\lambda \in {\mathbb {Z}}_{\ge 0}^{B^{(1)}}$ and $\gamma \in {\mathbb {Z}}_{\ge 0}^{B^{(2)}}$, we have that $\left\| \lambda \right\| _1, \left\| \gamma \right\| _1 \le (2d \Delta +1)^d$. Furthermore, the norm of *b* is bounded by $\left\| b\right\| _{\infty } \le \Delta (2d \Delta +1)^d$

#### Proof

Consider the representation of a point $b = B^{(1)} \lambda = B^{(2)} \gamma$ in the intersection of $\mathrm {int.cone}(B^{(1)})$ and $\mathrm {int.cone}(B^{(2)})$. The sum $v_1 + \cdots + v_{(\left\| \lambda \right\| _1 + \left\| \gamma \right\| _1)}$ consisting of $\lambda _i$ copies of the *i*th element of $B^{(1)}$ and $\gamma _i$ copies of the negative of the *i*th element of $B^{(2)}$ equals to zero. Using Steinitz’ Lemma, there exists a reordering of the vectors $u_1 + \cdots + u_{(\left\| \lambda \right\| _1 + \left\| \gamma \right\| _1)}$ such that the partial sum $\sum _{i=1}^\ell u_i \le d \Delta$, for each $\ell \le \left\| \lambda \right\| _1 + \left\| \gamma \right\| _1$.

If $\left\| \lambda \right\| _1 + \left\| \gamma \right\| _1 > (2d \Delta +1)^d$ then by the pigeonhole principle, there exist two partial sums of the same value. Hence, there are two sequences that sum up to zero, i.e., there exist non-zero vectors $\lambda ', \lambda '' \in {\mathbb {Z}}_{\ge 0}^{B^{(1)}}$ with $\lambda = \lambda ' + \lambda ''$ and $\gamma ', \gamma '' \in {\mathbb {Z}}_{\ge 0}^{B^{(2)}}$ with $\gamma = \gamma ' + \gamma ''$ such that $B^{(1)} \lambda ' - B^{(2)} \gamma '= 0$ and $B^{(1)} \lambda '' - B^{(1)} \gamma ''= 0$. Hence $B^{(1)} \lambda ' = B^{(2)} \gamma '$ and $B^{(1)} \lambda '' = B^{(2)} \gamma ''$ are elements of the intersection cone. This implies that *b* can be decomposed in the intersection cone. $\square$

Using a similar argumentation as in the previous lemma, we can consider the intersection of several integer cones. Note that we can not simply use the above Lemma inductively as this would lead to worse bounds.

#### Lemma 4

Consider integer cones $\mathrm {int.cone}(B^{(1)}), \ldots , \mathrm {int.cone}(B^{(\ell )})$ for some generating sets $B^{(1)}, \ldots , B^{(\ell )} \subset {\mathbb {Z}}_{\ge 0}^{d}$ with $\left\| x\right\| _{\infty } \le \Delta$ for each $x \in B^{(i)}$. Consider the integer cone of the intersection

$$\begin{aligned} \mathrm {int.cone}({\hat{B}}) = \bigcap _{i=1}^\ell \mathrm {int.cone}(B^{(i)}) \end{aligned}$$

for some generating set of elements ${\hat{B}}$.

Then for each indecomposable element $b \in {\hat{B}}$ with $B^{(i)} \lambda ^{(i)} = b$ for some $\lambda ^{(i)} \in {\mathbb {Z}}_{\ge 0}^{B^{(i)}}$ in the intersection cone, we have that $\left\| \lambda ^{(i)}\right\| _1 \le O((d \Delta )^{d (\ell -1)})$ for all $1 \le i \le \ell$.

#### Proof

Given vectors $\lambda ^{(1)}, \ldots , \lambda ^{(\ell )}$ with $\lambda ^{(k)} \in {\mathbb {Z}}_{\ge 0}^{B^{(k)}}$ and $B^{(k)} \lambda ^{(k)} = b$ for each $k \le \ell$. Consider the sum of vectors $v^{(k)}_1 + \cdots + v^{(k)}_{\left\| \lambda ^{(k)}\right\| _1}$ for each $1 \le k \le \ell$ consisting of $\lambda _j^{(k)}$ copies of the *j*th element of $B^{k}_j$. By adding 0 vectors to sums we can assume without loss of generality that every sequence has the same number of summands $L = \max _{i=1, \ldots , \ell } \left\| \lambda ^{(i)}\right\| _1$.

**Claim:** There exists a reordering $u^{(k)}_1 + \cdots + u^{(k)}_{L}$ for each of these sums such that each partial sum $p^{(k)}_m = \sum _{i =1}^m u^{(k)}_i$ is close to the line between 0 and *b* and more precisely:

$$\begin{aligned} \left\| p^{(k)}_m - \frac{m}{L} b\right\| _{\infty } \le 4 \Delta (d+1). \end{aligned}$$

for each $m \le L$ and each $k \le \ell$. To see this, we construct the sequence that consists of vectors from $B^{(k)}$ and subtract *L* fractional parts of the vector *b*. To count the number of vectors we use an additional component with weight $\Delta$ of the vector and define $${\bar{v}}^{(k)}_i = \begin{pmatrix} \Delta \\ v^{(k)}_i \end{pmatrix}$$ and $${\bar{b}} = \begin{pmatrix} L \Delta \\ b \end{pmatrix}$$. Note that $\left\| {\bar{v}}^{(k)}_i\right\| _1,\left\| \frac{1}{L}{\bar{b}}\right\| _1 \le 2 \Delta$. Then the sequence ${\bar{v}}^{(k)}_1 + \cdots + {\bar{v}}^{(k)}_{L} - \frac{1}{L} {\bar{b}} - \cdots - \frac{1}{L}{\bar{b}}$ sums up to zero, as $v^{(k)}_1 + \cdots + v^{(k)}_{L} = b$. Hence we can apply the Steinitz Lemma to obtain a reordering ${\bar{u}}_1 + \cdots + {\bar{u}}_L$ for each sequence such that each partial sum $\left\| \sum _{i=1}^m {\bar{u}}_i \right\| _{\infty } \le 2 \Delta (d+1)$ for each $m \le 2 L$. Each partial sum that sums up to index *m* contains *p* vectors ${\bar{v}}^{(k)}_j$ and *q* vectors $\frac{1}{L} b$ for some $p,q \in {\mathbb {Z}}_{\ge 0}$ with $m =p+q$. Hence $\sum ^{p}_{i=1} u_i - \frac{q}{L}b \le 2 \Delta (d+1)$. Furthermore, the $\Delta$ entry of each vector guarantees that $|p-q| \le 2(d+1)$ which implies the statement of the claim.

Now consider the differences of a partial sum $p^{(k)}_m$ with $p^{(1)}_m$. Using the claim from above, we can now argue that $\left\| p^{(1)}_m - p^{(k)}_m\right\| _{\infty } \le 8 \Delta (d+1)$ for each $m \le L$ and $k \le \ell$ as each $p^{(k)}_m$ is close to $\frac{m}{L}b$. Therefore the number of different values for $p^{(1)}_m - p^{(k)}_m$ is bounded by $(16 \Delta (d+1)+1)^d$. Assuming that $L > (16 \Delta (d+1)+1)^{d(\ell -1)}$, by the pigeonhole principle there exist indices $m'$ and $m''$ with $m' > m''$ such that $p^{(1)}_{m'} - p^{(k)}_{m'} = p^{(1)}_{m''} - p^{(k)}_{m''}$ for each $k \le \ell$. Hence $p^{(1)}_{m'} - p^{(1)}_{m''} = \ldots = p^{(\ell )}_{m'} - p^{(\ell )}_{m''} =: b'$ and $b', b-b' \in \cap _{i=1}^\ell B_i$. This implies that *b* can be decomposed and is therefore not a generating element of $\cap _{i=1}^\ell \mathrm {int.cone}(B^i).$ $\square$

### Proof of Lemma

2 Using the results from the previous section, we are now finally able to prove the main Lemma 2.

We begin with a sketch of the proof for the 1-dimensional case. This will be helpful when we generalize the approach later. In the 1-dimensional case, the multisets $T_i$ consist solely of natural numbers, i.e $T_1, \ldots , T_n \subset {\mathbb {Z}}_{\ge 0}$. Suppose that each set $T_i$ consists only of many copies of a single integral number $x_i \in \{1 , \ldots , \Delta \}$. Then it is easy to find a common multiple as $\frac{\Delta !}{1} \cdot 1 = \frac{\Delta !}{2} \cdot 2 = \ldots = \frac{\Delta !}{\Delta } \cdot \Delta$. Hence one can choose the subsets consisting of $\frac{\Delta !}{x_i}$ copies of $x_i$. Now suppose that the multisets $T_i$ can be arbitrary. If $|T_i| \le \Delta \cdot \Delta ! = \Delta ^{O(\Delta )}$ we are done. But on the other hand, if $|T_i| \ge \Delta \cdot \Delta !$, by the pigeonhole principle there exists a single element $x_i \in \{1, \ldots , \Delta \}$ for every $T_i$ that appears at least $\Delta !$ times. Then we can argue as in the previous case where we needed at most $\Delta !$ copies of a number $x_i \in \{ 1, \ldots , \Delta \}$. Note that the cardinality of the sets $T_i$ has to be of similar size. As the elements of each set sums up to the same value, the cardinality of two sets $T_i, T_j$ can only differ by a factor of $\Delta$. This proves the lemma in the case $d=1$.

In the case of higher dimensions, the lemma seems much harder to prove. But in principle we use generalizations of the above techniques. Instead of single natural numbers however, we have to work with bases of corresponding basic feasible LP solutions and the intersections of the integer cones generated by those bases.

In the proof we need the notion of a cone which is simply the relaxation of an integer cone. For a generating set $B \subset {\mathbb {Z}}_{\ge 0}^d$, a *cone* is defined by

$$\begin{aligned} cone(B) = \left\{ \sum _{b \in B} \lambda _b b \mid \lambda \in {\mathbb {R}}_{\ge 0}^B\right\} . \end{aligned}$$

#### Proof

First, we describe the multisets $T_1, \ldots , T_n \subset {\mathbb {Z}}_{\ge 0}^d$ by multiplicity vectors $\lambda ^{(1)}, \ldots , \lambda ^{(n)} \in {\mathbb {Z}}_{\ge 0}^{P}$, where $P \subset {\mathbb {Z}}^d$ is the set of non-negative integer points *p* with $\left\| p\right\| _{\infty } \le \Delta$. Each $\lambda ^{(i)}_{p}$ thereby states the multiplicity of a vector *p* in $T_i$. Hence $\sum _{t \in T_i} t = \sum _{p \in P} \lambda ^{(i)}_{p} p$ and our objective is to find vectors $y^{(1)}, \ldots , y^{(n)} \in {\mathbb {Z}}_{\ge 0}^P$ with $y^{(i)} \le \lambda ^{(i)}$ such that $\sum _{p \in P} y^{(1)}_{p} p = \ldots = \sum _{p \in P} y^{(n)}_{p} p$.

Consider the linear program

3 $$\begin{aligned}&\sum _{p \in P} x_p p = b\nonumber \\&x \in {\mathbb {R}}_{\ge 0}^P \end{aligned}$$

Let $x^{(1)}, \ldots , x^{(\ell )} \in {\mathbb {R}}_{\ge 0}^d$ be all possible basic feasible solutions of the LP corresponding to bases $B^{(1)}, \ldots , B^{(\ell )} \in {\mathbb {Z}}_{\ge 0}^{d \times d}$ i.e., $B^{(i)} x^{(i)} = b$.

In the following we prove two claims that correspond to the two previously described cases of the one dimensional case. First, we consider the case that essentially each multiset $T_i$ corresponds to one of the basic feasible solution $x^{(j)}$. In the 1-dimensional case this would mean that each set consists only of a single number. Note that the intersection of integer cones in dimension 1 is just the least common multiple, i.e., $\mathrm {int.cone}(z_1) \cap \mathrm {int.cone}(z_2) = \mathrm {int.cone}(\mathrm {lcm}(z_1, z_2))$ for some $z_1, z_2 \in {\mathbb {Z}}_{\ge 0}$.

#### Claim 1

If for all *i* we have $\left\| x^{(i)}\right\| _1 > d \cdot O((d \Delta )^{d (\ell -1)})$ then there exist non-zero vectors $y^{(1)}, \ldots , y^{(\ell )} \in {\mathbb {Z}}_{\ge 0}^d$ with $y^{(1)} \le x^{(1)}, \ldots , y^{(\ell )} \le x^{(\ell )}$ and $\left\| y^{(i)}\right\| _1 \le d \cdot O((d \Delta )^{d (\ell -1)})$ such that $B^{(1)}y^{(1)} = \ldots = B^{(\ell )} y^{(\ell )}$.

Note that all basic feasible solutions $x^{(i)} \in {\mathbb {R}}^{d}_{\ge 0}$ have to be of similar size. Since $B x^{(i)} = b$ holds for all $1 \le i \le \ell$ we know that $\left\| x^{(i)}\right\| _1$ and $\left\| x^{(j)}\right\| _1$ can only differ by a factor of $d \Delta$ for all $1 \le i,j \le \ell$. Hence all basic feasible solutions $x^{(i)}$ have to be either small or all have to be large. This claim considers the case that the size of all $x^{(i)}$ is large.

#### Proof of the claim

Note that $B^{(i)} x^{(i)} = b$ and hence $b \in cone(B^{(i)})$. In the following, our goal is to find a non-zero point $q \in {\mathbb {Z}}_{\ge 0}^d$ such that $q = B^{(1)} y^{(1)} = \ldots = B^{(\ell )} y^{(\ell )}$ for some vectors $y^{(1)}, \ldots , y^{(\ell )} \in {\mathbb {Z}}_{\ge 0}^d$. However, this means that *q* has to be in the integer cone $\mathrm {int.cone}(B^{(i)})$ for every $1 \le i \le \ell$ and therefore in the intersection of all the integer cones, i.e., $q \in \bigcap _{i=1}^n \mathrm {int.cone}(B^{(i)})$. By Lemma 4 there exists a set of generating elements ${\hat{B}}$ such that

- $\mathrm {int.cone}({\hat{B}}) = \bigcap _{i=1}^n \mathrm {int.cone}(B^{(i)})$ and $\mathrm {int.cone}({\hat{B}}) \ne \{ 0 \}$ as $b \in cone({\hat{B}})$ and
- each generating vector $p \in {\hat{B}}$ can be represented by $p = B^{(i)} \lambda$ for some $\lambda \in {\mathbb {Z}}_{\ge 0}^d$ with $\left\| \lambda \right\| _1 \le O((d \Delta )^{d (\ell -1)})$ for each basis $B^{(i)}$.

As $b \in \mathrm {cone}({\hat{B}})$ there exists a vector ${\hat{x}} \in {\mathbb {R}}_{\ge 0}^{{\hat{B}}}$ with ${\hat{B}} {\hat{x}} = b$. Our goal is to show that there exists a non-zero vector $q \in {\hat{B}}$ with ${\hat{x}}_q \ge 1$. In this case *b* can be simply written by $b = q + q'$ for some $q' \in \mathrm {cone}({\hat{B}})$. As *q* and $q'$ are contained in the intersection of all cones, there exists for each generating set $B^{(j)}$ a vectors $y^{(j)} \in {\mathbb {Z}}_{\ge 0}^{B^{(j)}}$ and $z^{(j)} \in {\mathbb {R}}_{\ge 0}^{B^{(j)}}$ such that $B^{(j)} y^{(j)} = q$ and $B^{(j)} z^{(j)} = q'$. Hence $x^{(j)} = y^{(j)} + z^{(j)}$ and we finally obtain that $x^{(j)} \ge y^{(j)}$ for $y^{(j)} \in {\mathbb {Z}}_{\ge 0}^{B^{(j)}}$ which shows the claim.

Therefore it only remains to prove the existence of the point *q* with ${\hat{x}}_q \ge 1$. By Lemma 4, each vector $p \in {\hat{B}}$ can be represented, by $p = B^{(i)} x^{(p)}$ for some $x^{(p)} \in {\mathbb {Z}}_{\ge 0}^{B^{(i)}}$ with $\left\| x^{(p)}\right\| _1 \le O((d \Delta )^{d (\ell -1)})$ for every basis $B^{(i)}$.

As $B^{(i)} x^{(i)} = b = \sum _{p \in {\hat{B}}} {\hat{x}}_p p = \sum _{p \in {\hat{B}}} {\hat{x}}_p (B^{(i)} x^{(p)})$, every $x^{(i)}$ can be written by $x^{(i)} = \sum _{p \in {\hat{B}}} x^{(p)} {\hat{x}}_p$ and we obtain a bound on $\left\| x^{(i)}\right\| _1$ assuming that every for every $p \in {\hat{B}}$ we have ${\hat{x}}_p < 1$.

$$\begin{aligned} \left\| x^{(i)}\right\| _1 \le \sum _{p \in {\hat{B}}} \left\| x^{(p)} {\hat{x}}_p\right\| _1 {\mathop {<}\limits ^{{\hat{x}}_p < 1}} \sum _{p \in {\hat{B}}} \left\| x^{(p)}\right\| _1 \le d \cdot O((d \Delta )^{d (\ell -1)}). \end{aligned}$$

The last inequality follows as we can assume by Caratheodory’s theorem [30] that the number of non-zero components of ${\hat{x}}$ is less or equal than *d*. Hence if $\left\| x^{(i)}\right\| _1 \ge d \cdot O((d \Delta )^{d (\ell -1)})$ then there has to exist a vector $q \in {\hat{B}}$ with $x_q \ge 1$ which proves the claim. $\square$

#### Claim 2

For every vector $\lambda ^{(i)} \in {\mathbb {Z}}_{\ge 0}^P$ with $\sum _{p \in P} \lambda _p p = b$ there exists a basic feasible solution $x^{(k)}$ of LP (3) with basis $B^{(k)}$ such that $\frac{1}{\ell }x^{(k)} \le \lambda ^{(i)}$ in the sense that $\frac{1}{\ell } x^{(k)}_p \le \lambda ^{(i)}_p$ for every $p \in B^{(k)}$.

#### Proof of the claim

The proof of the claim can be easily seen as each multiplicity vector $\lambda ^{(i)}$ is also a solution of the linear program (3). By standard LP theory, we know that each solution of the LP is a convex combination of the basic feasible solutions $x^{(1)}, \ldots , x^{(\ell )}$. Hence, each multiplicity vector $\lambda ^{(i)}$ can be written as a convex combination of $x^{(1)}, \ldots , x^{(\ell )}$, i.e., for each $\lambda ^{(i)}$, there exists a $t \in {\mathbb {R}}_{\ge 0}^\ell$ with $\left\| t\right\| _1 = 1$ such that $\lambda ^{(i)} = \sum _{j=1}^\ell t_j {\bar{x}}^{(j)}$, where

$$\begin{aligned} {\bar{x}}^{(j)}_p~=~{\left\{ \begin{array}{ll} x^{(j)}_p &{}\text { if } p \in B^{(j)} \\ 0 &{} \text { otherwise}\end{array}\right. }. \end{aligned}$$

By the pigeonhole principle, there exists for each multiplicity vector $\lambda ^{(i)}$ an index *k* with $t_k \ge \frac{1}{\ell }$ which proves the claim. $\square$

Using the above two claims, we can now prove the claim of the lemma by showing that for each $\lambda ^{(i)}$, there exist a vector $y^{(i)} \le \lambda ^{(i)}$ with bounded 1-norm such that $\sum _{p \in P} y^{(1)}_p p = \ldots = \sum _{p \in P} y^{(n)}_p p$.

First, consider the case that there exists a basic feasible solution $x^{(j)}$ of LP 3 with $\left\| x^{(j)}\right\| _1 \le \ell d \cdot O((d \Delta )^{d (\ell -1)})$. In this case we have for all $1 \le i \le n$ that $\left\| \lambda ^{(i)}\right\| _1 \le \ell d^2 \Delta \cdot O((d \Delta )^{d (\ell -1)})$ as the size of solutions of LP (3) can not differ by a factor of more than $d \Delta$ (this is because for every $p,p' \in P$ the sizes $\left\| p\right\| _1, \left\| p'\right\| _1$ can not differ by a factor of more than $d \Delta$).

Now, assume that for all basic feasible solutions $x^{(i)}$ we have $\left\| x^{(i)}\right\| _1 > \ell d \cdot O((d \Delta )^{d (\ell -1)})$. We can argue by Claim 2 that for each $\lambda ^{(i)}$ (with $1 \le i \le n$) we find one of the basic feasible solutions $x^{(k)}$ ($1 \le k \le \ell$) with $\frac{1}{\ell } x^{(k)} \le \lambda ^{(i)}$. As $\frac{1}{\ell } x^{(i)} \ge d \cdot O((d \Delta )^{d (\ell -1)})$ for all $1 \le i \le \ell$, we can apply the first claim to vectors $\frac{1}{\ell } x^{(1)}, \ldots , \frac{1}{\ell } x^{(\ell )}$ with $\frac{1}{\ell }b = \frac{1}{\ell } Bx^{(1)} = \ldots = \frac{1}{\ell } B x^{(\ell )}$, we obtain vectors $y^{(1)} \le \frac{1}{\ell }x^{(1)}, \ldots , y^{(\ell )} \le \frac{1}{\ell }x^{(\ell )}$ with $By^{(1)} = \ldots = B y^{(\ell )}$. Hence, we find for each $\lambda ^{(i)}$ a vector $y^{(k)} \in {\mathbb {Z}}_{\ge 0}^{B^{(k)}}$ with $y^{(k)} \le \lambda ^{(i)}$.

Finally we obtain that

$$\begin{aligned} \left\| y^{(j)}\right\| _1 \le d^2 \Delta \ell \cdot O((d \Delta )^{d (\ell -1)}) = (d \Delta )^{O(d (\Delta ^{d^2}))} \end{aligned}$$

using that $\ell$ is bounded by $$\left( {\begin{array}{c}|P|\\ d\end{array}}\right) \le |P|^d$$ and $|P| \le \Delta ^d$ $\square$

## A lower bound for the size of graver elements

In this section we prove a lower bound on the size of Graver elements for a matrix where the overlapping parts contains only a single variable, i.e., $s=1$.

First, consider the matrix

$$\begin{aligned} {\mathcal {A}} = \begin{pmatrix} -1 &{} 2 &{} 0 &{} \cdots &{} 0\\ -1 &{} 0 &{} 3 &{} \ddots &{} \vdots \\ -1 &{} \vdots &{} \ddots &{} \ddots &{} 0\\ -1 &{} 0 &{} \cdots &{} 0 &{} M\\ \end{pmatrix}. \end{aligned}$$

This matrix is of 2-stage stochastic structure with $r=1$ and $s=1$. We will argue that every element in $\mathrm {ker}({\mathcal {A}}) \cap ({\mathbb {Z}}^M {\setminus } \{ 0 \})$ is large and therefore, the Graver elements of the matrix are large as well. We call the variable corresponding to the *i*th column of the matrix variable $x_i$, where $x_1$ is the variable corresponding to the column with only $-1$ entries and the $x_i$ for $i>1$ is the variable corresponding to the column with entry *i* in component *i* and 0 everywhere else. Clearly, for $x \in {\mathbb {Z}}^{M}$ to be in $\mathrm {ker}({\mathcal {A}}) \cap {\mathbb {Z}}^n$, we know by the first row of matrix ${\mathcal {A}}$ that $x_1$ has to be a multiple of 2. By the second row of the matrix, we know that $x_1$ has to be a multiple of 3 and so on. Henceforth the variable $x_1$ has to be a multiple of all numbers $1, \ldots , M$. Thus $x_1$ is a multiple of the least common multiple of numbers $1, \ldots , n$ which is divisible by the product of all primes between $1, \ldots , n$. By known bounds for the product of all primes $\le n$ [13], this implies that the value of $x_1 \in 2^{\Omega (M)}$, which shows that the size of Graver elements of matrix ${\mathcal {A}}$ is in $(2^{\Omega (M)})$.

The disadvantage in the matrix above is that the entries of the matrix are rather big. In the following we reduce the largest entry of the overall matrix by encoding each number $1, \ldots , M$ into a submatrix. For the encoding we use the matrix

$$\begin{aligned} {\mathcal {C}} = \begin{pmatrix} \Delta &{} - 1 &{} 0 &{} \cdots &{} 0 \\ 0 &{} \Delta &{} -1 &{} \ddots &{} \vdots \\ \vdots &{} \ddots &{} \ddots &{} \ddots &{} 0\\ 0 &{} \cdots &{} 0 &{} \Delta &{} -1 \end{pmatrix}, \end{aligned}$$

having *r* rows and $r+1$ constraints. Due to the first row of matrix ${\mathcal {C}}$, for a vector $x \in \mathrm {ker}({\mathcal {C}}) \cap {\mathbb {Z}}^{r+1}$ we know by the *i*th row of the matrix that $x_i= x_{i-1} \cdot \Delta$. Hence $x_i = \Delta ^{i-1} x_1$. Now we can encode in each number $z \in \{ 0, \ldots , \Delta ^{r+1} -1 \}$ in an additional row by $z = \sum _{i=0}^{r} a_i(z) \Delta ^i$, where $a_i(z)$ is the *i*th number in a representation of *z* in base $\Delta$. Hence, we consider the following matrix:By the same argumentation as in matrix ${\mathcal {A}}$ above we know that $x_0$ has to be a multiple of each number $2, \ldots ,\Delta ^{r+1}-1$. This implies that every non-zero integer vector of $\mathrm {ker}({\mathcal {A}}')$ has infinity-norm of at least $2^{\Omega (\Delta ^{r})}$. This shows the doubly exponential lower bound for the Graver complexity of 2-stage stochastic IPs and proves the following theorem.

### Theorem 4

There exists a constraint matrix ${\mathcal {A}} \in {\mathbb {Z}}^{rn \times (1+nt)}$ such that each Graver element $y \in \mathrm {ker}({\mathcal {A}})$ is lower bounded by

$$\begin{aligned} \left\| y\right\| _{\infty } \ge 2^{\Omega (\Delta ^r)}. \end{aligned}$$

## Multi-stage stochastic IPs

In this section we show that Lemma 2 can also be used to get a bound on the Graver elements of matrices with a multi-stage stochastic structure. Multi-stage stochastic IPs are a well known generalization of 2-stage stochastic IPs. For the stochastical programming background on multi-stage stochastic IPs we refer to [29]. Here we simply show how to solve the equivalent deterministic IP with a large constraint matrix. Regarding the augmentation framework of multi-stage stochastic IPs, it was previously known that a similar implicit bound as for 2-stage stochastic IPs also holds for multi-stage stochastic IPs. This was shown by Aschenbrenner and Hemmecke [2] who built upon the bound of 2-stage stochastic IPs.

In the following we define the shape of the constraint matrix ${\mathcal {M}}$ of a multi-stage stochastic IP. The constraint matrix consists of given matrices $A^{(1)}, \ldots , A^{(\ell )}$ for some $\ell \in {\mathbb {Z}}_{\ge 0}$, where each matrix $A^{(i)}$ uses a unique set of columns in ${\mathcal {M}}$. For a given matrix, let $\mathrm {rows}(A^{(i)})$ be the set of rows in ${\mathcal {M}}$ which are used by $A^{(i)}$. A matrix ${\mathcal {M}}$ is multi-stage stochastic shape, if the following conditions are fulfilled:

- There is a matrix $A^{i_0}$ such that for every $1 \le i \le n$ we have $\mathrm {rows}(A^{(i)}) \subseteq \mathrm {rows}(A^{(i_0)})$.
- For two matrices $A^{(i)}, A^{(j)}$ either $\mathrm {rows}(A^{(i)}) \subseteq \mathrm {rows}(A^{(j)})$ or $\mathrm {rows}(A^{(i)}) \cap \mathrm {rows}(A^{(j)}) = \emptyset$ holds.

An example of a matrix of multi-stage stochastic structure is given in the following: 
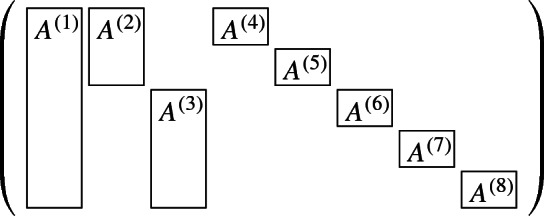


Intuitively, the constraint matrix is of multi-stage stochastic shape if the block matrices with the relation $\subseteq$ on the rows, forms a tree (see figure below). 
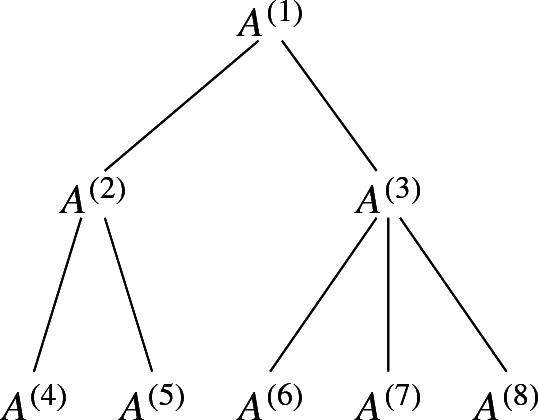
 Let $s_i$ be the number of columns that are used by matrices in the *i*th level of the tree (starting from level 0 at the leaves). Here we assume that the number of columns of matrices in the same level of the tree are all identical. Let *r* be the number of rows that are used by the matrices that correspond to the leaves of the tree. In the following theorem we show that Lemma 2 can be applied inductively to bound the size of an augmenting step of multi-stage stochastic IPs. The proof is similar to that of Theorem 2.

### Theorem 5

Let *y* be an indecomposable cycle of matrix ${\mathcal {M}}$. Then $\left\| y\right\| _{\infty }$ is bounded by a function $T(s_1, \ldots , s_{t},r, \Delta )$, where *t* is the depth of the tree. The function *T* involves a tower of $t+1$ exponentials and is recursively defined by

$$\begin{aligned}&T(r, \Delta ) = (\Delta r)^{O(r)}\\&T(s_1, \ldots , s_i,r, \Delta ) = 2^{(T(s_1, \ldots , s_{i-1},r, \Delta ))^{O(s^{2}_i)}}. \end{aligned}$$

### Proof

Consider a submatrix ${\mathcal {A}}$ of the constraint matrix ${\mathcal {M}}$ corresponding to a subtree of the tree with depth *t*. Hence, ${\mathcal {A}}$ itself is of multi-stage stochastic structure. Let submatrix $A \in \{A^{(1)}, \ldots , A^{(\ell )} \}$ be the root of the corresponding subtree of ${\mathcal {A}}$ and let the submatrices $B^{(1)}, \ldots , B^{(n)}$ be the submatrices corresponding to the subtrees of *A* with $\mathrm {rows}(B^{(i)}) \subseteq \mathrm {rows}(A)$ for all $1 \le i \le n$.

Let ${\bar{A}}^{(i)}$ be the submatrix of *A* which consists only of the rows that are used by $B^{(i)}$ (recall that $\mathrm {rows}(B^{(i)}) \subseteq \mathrm {rows}(A)$). Now suppose that *y* is a cycle of ${\mathcal {A}}$, i.e., ${\mathcal {A}} y =0$ and let $y^{(0)}$ be the subvector of *y* consisting only of the entries that belong to matrix *A*. Symmetrically let $y^{(i)}$ be the entries of vector *y* that belong only to the matrix $B^{(i)}$ for $i>0$. Since ${\mathcal {A}}y = 0$ we also know that $${\bar{A}}^{(i)}y^{(0)} + B^{(i)}y^{(i)} = ({\bar{A}}^{(i)} B^{(i)}) \begin{pmatrix} y^{(0)} \\ y^{(i)} \end{pmatrix}= 0$$ for every $1 \le i \le n$. Each vector $$\begin{pmatrix} y^{(0)} \\ y^{(i)} \end{pmatrix}$$ can be decomposed into a multiset of indecomposable cylces $C_i$ , i.e.,

$$\begin{aligned} \begin{pmatrix} y^{(0)} \\ y^{(i)} \end{pmatrix} = \sum _{z \in C_i} z \end{aligned}$$

where each cycle $z \in C_i$ is a vector $$z = \begin{pmatrix} z^{(0)} \\ z^{(i)} \end{pmatrix}$$ consisting of subvector $z^{(0)}$ of entries that belong to matrix *A* and a subvector $z^{(i)}$ of entries that belong to the matrix $B^{(i)}$. Note that the matrix $(A^{(i)} \, B^{(i)})$ has a multi-stage stochastic structure with a corresponding tree of depth $t-1$. Hence, by induction we can assume that each indecomposable cycle $z \in C_i$ is bounded by $\left\| z\right\| _{\infty } \le T(s_1, \ldots , s_{t-1}, r)$ for all $1 \le i \le n$, where *T* is a function that involves a tower of *t* exponentials. In the base case that $t=0$ and the matrix ${\mathcal {A}}$ only consists of one matrix, we can bound $\left\| z\right\| _{\infty }$ by $(2 \Delta r+1)^r$ using Theorem 1. Let *p* be the projection that maps a cycle to the entries that belong to the matrix *A* i.e., $$p(z) = p(\begin{pmatrix} z^{(0)} \\ z^{(i)} \end{pmatrix}) = z^{(0)}$$.

For each vector $$\begin{pmatrix} y^{(0)} \\ y^{(i)} \end{pmatrix}$$ and its decomposition into cycles $C_i$ let $p(C_i) = \{ p(z) \mid z \in C_i \}$. Since

$$\begin{aligned} y^{(0)} = \sum _{z \in C_1} p(z) = \ldots = \sum _{z \in C_n} p(z) \end{aligned}$$

we can apply Lemma 2, to obtain submultisets $S_i \subseteq p(C_i)$ of bounded size

$$\begin{aligned} |S_i| \le (s_t T)^{O(s_t (T^{s_{t}^2}))} \end{aligned}$$

with $T = T(s_1, \ldots , s_{t-1}, r, \Delta )$ such that $\sum _{x \in S_1}x = \ldots = \sum _{x \in S_n} x$. As $T(s_1, \ldots , s_{t-1}, r)$ is a function with *t* exponentials, the cardinality $|S_i|$ can be bounded by a function of $t+1$ exponentials.

There exist submultisets ${\bar{C}}_1 \subseteq C_1, \ldots , {\bar{C}}_n \subseteq C_n$ with $p({\bar{C}}_1) = S_1, \ldots , p({\bar{C}}_n) = S_n$. Hence, we can define the solution ${\bar{y}} \le y$ by ${\bar{y}}^{(i)} = \sum _{z \in {\bar{C}}_i} {\bar{p}}(z)$ for every $i>0$, where ${\bar{p}}$ is the function that projects a vector to the entries that belong the matrix $B^{(i)}$ i.e., $${\bar{p}}(z) = {\bar{p}}(\begin{pmatrix} z^{(0)} \\ z^{(i)} \end{pmatrix}) = z^{(i)}$$. For $i=0$ we define $y^{(0)} = \sum _{z \in {\bar{C}}_i} p(z)$. As the sum $\sum _{z \in {\bar{C}}_i} p(z)$ is identical for every $1 \le i \le n$, the vector ${\bar{y}}$ is a well defined.

Let *K* be the constant derived from the *O*-notation of Lemma 2 and $T = T(s_1, \ldots , s_{t-1}, r, \Delta )$, then the size of ${\bar{y}}$ can be bounded by

$$\begin{aligned} \left\| {\bar{y}}\right\| _{\infty } \le T \cdot \max _i |C_i| = T \cdot (s_t T)^{K s_t \cdot T^{(s^{2}_t)}} \le 2^{K s_t \log (s_t T) \cdot T^{(s^{2}_t)}} \le 2^{T^{O(s^{2}_t)}}. \end{aligned}$$

$$\square $$

As a consequence of the bound of the Graver elements of the constraint matrix ${\mathcal {M}}$ of multi-stage stochastic IPs, we obtain by using the augmentation framework an algorithm to solve multi-stage stochastic IPs. Again, we refer to [11] for the details on the augmentation framework.

### Theorem 6

A multi-stage stochastic IP with a constraint matrix ${\mathcal {M}}$ that corresponds to a tree of depth *t* can be solved in time

$$\begin{aligned} n^2s_{0}^2 \varphi \log ^2 (n s_0) \cdot T(s_1, \ldots , s_{t}, r, \Delta ), \end{aligned}$$

where $\varphi$ is the encoding length of the IP and *T* is a function depending only on parameters $s_1, \ldots , s_t,r, \Delta$ and involves a tower of $t+1$ exponentials.
